# Emergence of Serotonergic Neurons After Spinal Cord Injury in Turtles

**DOI:** 10.3389/fncir.2018.00020

**Published:** 2018-03-13

**Authors:** Gabriela Fabbiani, María I. Rehermann, Carina Aldecosea, Omar Trujillo-Cenóz, Raúl E. Russo

**Affiliations:** Departamento de Neurofisiología Celular y Molecular, Instituto de Investigaciones Biológicas Clemente Estable (IIBCE), Montevideo, Uruguay

**Keywords:** spinal cord injury, plasticity, neurotransmitter respecification, serotonin, non-mammalian vertebrates

## Abstract

Plasticity of neural circuits takes many forms and plays a fundamental role in regulating behavior to changing demands while maintaining stability. For example, during spinal cord development neurotransmitter identity in neurons is dynamically adjusted in response to changes in the activity of spinal networks. It is reasonable to speculate that this type of plasticity might occur also in mature spinal circuits in response to injury. Because serotonergic signaling has a central role in spinal cord functions, we hypothesized that spinal cord injury (SCI) in the fresh water turtle *Trachemys scripta elegans* may trigger homeostatic changes in serotonergic innervation. To test this possibility we performed immunohistochemistry for serotonin (5-HT) and key molecules involved in the determination of the serotonergic phenotype before and after SCI. We found that as expected, in the acute phase after injury the dense serotonergic innervation was strongly reduced. However, 30 days after SCI the population of serotonergic cells (5-HT+) increased in segments caudal to the lesion site. These cells expressed the neuronal marker HuC/D and the transcription factor Nkx6.1. The new serotonergic neurons did not incorporate the thymidine analog 5-bromo-2′-deoxyuridine (BrdU) and did not express the proliferating cell nuclear antigen (PCNA) indicating that novel serotonergic neurons were not newborn but post-mitotic cells that have changed their neurochemical identity. Switching towards a serotonergic neurotransmitter phenotype may be a spinal cord homeostatic mechanism to compensate for the loss of descending serotonergic neuromodulation, thereby helping the outstanding functional recovery displayed by turtles. The 5-HT_1A_ receptor agonist (±)-8-Hydroxy-2-dipropylaminotetralin hydrobromide (8-OH-DPAT) blocked the increase in 5-HT+ cells suggesting 5-HT_1A_ receptors may trigger the respecification process.

## Introduction

Traumatic spinal injury in adult mammals leads to chronic paralysis due to both the interruption of descending motor commands and impaired neuromodulation of spinal circuits (Hagen, 2015). However, some non-mammalian vertebrates show a substantial degree of functional recovery because they are endowed with self-repair mechanisms (Tanaka and Ferretti, 2009). In fresh water turtles, a cellular bridge reconnects the transected spinal cord allowing substantial recovery of stepping locomotion (Rehermann et al., 2009, 2011).

Locomotion depends on three main control levels: sensory afferents, spinal central pattern generators (CPGs) and extrinsic supraspinal commands. Spinal motor systems are modified by both intrinsic and extrinsic neuromodulation, which allows adaptation to diverse physiological situations (Marder, 2012). A key regulation of the CPGs is exerted by serotonin (5-HT) released from descending fibers which promotes the generation of rhythmic motor activity in the absence of sensory stimuli (Schotland and Grillner, 1993). In turtles, the source of the serotonergic innervation of the whole central nervous system (CNS) arises mostly from serotonergic neurons localized in caudal raphe nuclei of the brainstem (Kiehn et al., 1992). 5-HT+ fibers run in the lateral funiculus from which collateral fibers and synaptic buttons spread all over the spinal cord invading particularly the motor nuclei (Kiehn et al., 1992). Spinal motoneurons (Mns) express 5-HT receptors (5-HTr) 1A, B, D, 2A-C and 5A whose topology determine their firing output (Perrier et al., 2013).

The interruption of descending fibers by a lesion leads to the loss of serotonergic innervation with the disruption of the neuromodulatory tone. It has been proposed that homeostatic changes in spinal circuits below the lesion may be a major component of functional recovery (Rossignol et al., 2007). For example, spinal cord injury (SCI) in rats shifts 5-HTr subtype 2C towards a constitutively active form (Chanrion et al., 2008), increasing the excitability of Mns caudal to the lesion site and improving locomotor performance (Fouad et al., 2010). The exogenous application of 5-HT or its precursor 5-Hydroxytryptophan (5-HTP) as well as 5-HTr agonists in spinal cats facilitates locomotion recovery by means of the CPG activation (Brustein and Rossignol, 1999). These studies suggest that a critical step for functional recovery would be the restoration of serotonergic innervation beyond the lesion site. Interestingly, in organotypic cultures of the spinal cord of mouse embryos the decrease of serotonergic fibers is followed by the appearance of new 5-HT+ cells in the ventral gray (Branchereau et al., 2002). We speculate that the upregulation of the intrinsic serotonergic innervation may occur in turtles after SCI to compensate the lack of serotonergic fibers from supraspinal levels.

To test this hypothesis, we transected the cord at low thoracic levels and performed immunohistochemistry for 5-HT in the lumbar spinal cord at different times after injury. We found that the dense network of serotonergic fibers disappeared but a new set of 5-HT+ cells appeared in the lumbar-ventral cord. These novel 5-HT+ cells expressed neuronal markers and some transcription factors (TF) involved in determining the serotonergic neurochemical phenotype. Injured turtles treated with the selective 5-HT_1A_r agonist (±)-8-Hydroxy-2-dipropylaminotetralin hydrobromide (8-OH-DPAT) showed a lower number of 5-HT+ cells than non-treated animals, suggesting that 5-HT_1A_r are implied in neurotransmitter respecification after injury.

## Materials and Methods

### Animals

Fresh-water turtles (*Trachemys scripta elegans*) with carapace lengths ranging between 5 cm and 12 cm were maintained in temperate aquaria (28°C) and artificially illuminated in a 12 h light—12 h dark cycle. These juvenile turtles behave like fully mature animals in terms of their sensory-motor behavior. This study was carried out in accordance with the recommendations of the local National Committee for Animal Research (CNEA). The protocol (#005-09-2015) was approved by our Institutional Ethics Committee for Animal Use (CEUA).

### Surgical Procedures

**Figure 1:**
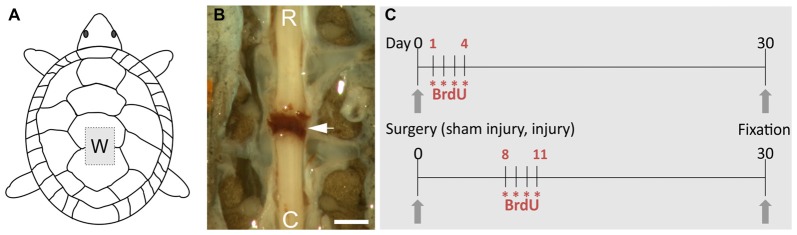
Overview of the procedures to study spinal cord injury (SCI) in the turtle. **(A)** Schematic drawing showing the “window” made in the carapace (W) to transect the spinal cord. **(B)** Image of the spinal cord after transection, the arrow indicates the clot linking the separated spinal stumps (R, rostral; C, caudal). Scale bar = 1 mm. **(C)** Schemes of the 5-bromo-2′-deoxyuridine (BrdU) injection protocols. Each asterisk represents two daily injections of BrdU.

To transect the spinal cord, the animals were first sedated with ketamine chlorhydrate (40 mg/kg b.w. intraperitoneal injection). Then, the four legs were secured to a rigid support by means of rubber bands and complete anesthesia was achieved by inhalation of Isoflurane (LIBRA, Minrad INC, PA, USA). To prevent infection, we rubbed the carapace with a cotton pad immersed in an alcohol-iodine solution and the surgical instruments were treated with 75% ethanol. After the animals were unresponsive to nociceptive stimuli, we opened a window in the carapace centered in the junction between the third and fourth dorsal scutes to expose the low thoracic spinal cord. Complete transection of the spinal cord was performed between dorsal segments 5 and 6 (equivalent to mammalian low thoracic segments, Mortin and Stein, 1990) with a thin-blade scalpel. Transection produced the retraction and separation of both stumps with the space filled with a blood clot (Figure 1). After washing with sterile saline the lifted scutes were re-placed and their borders sealed with cyanoacrylate (Rehermann et al., 2009, 2011). Sham-injured (ShI) animals were manipulated as described above, exposing the spinal cord but without any further action.

### Post-Operative Care

After recovery from anesthesia the animals were able to swim using their forelimbs and eat normally, not requiring special post-operative care despite the lack of spontaneous mobility of their hind limbs. The same post-surgery procedures were employed when dealing with ShI animals. We studied a total of 22 turtles (spinal cord-injured [ScI] = 16; ShI = 6) whose spinal cords were fixed at the following time intervals after transection: 30 days (ScI = 6; ShI = 6) and 10 days (ScI = 10, from which six were treated with 5-HT agonist 8-OH-DPAT and the remaining four received saline).

### 5-Bromo-2′-Deoxyuridine (BrdU) Injections

ScI and ShI turtles were both injected with 5-bromo-2′-deoxyuridine (BrdU; SIGMA) in order to identify newly generated cells. Two daily injections of 100 mg/kg (i.p.) were applied during four consecutive days starting 24 h (ShI = 3, ScI = 3) or 1 week (ShI = 3, ScI = 3) after surgery. The animals were fixed at day 30 (see Figure 1C).

### (±)-8-Hydroxy-2-Dipropylaminotetralin Hydrobromide (8-OH-DPAT) Injections

In six ScI animals the 5-HT_1A_r agonist 8-OH-DPAT (TOCRIS) was injected twice a day during 10 days (0.5 mg/kg, i.p.) and 12 h after the last injection, the animals were perfused and processed for immunohistochemical detection of 5-HT.

### General Histological Procedures

In all experimental protocols the spinal cord of anesthetized turtles (60 mg/kg sodium pentobarbital, i.p.) was fixed by perfusion through the heart 10 or 30 days after surgery. For immunohistochemistry, we used different paraformaldehyde concentrations to optimize preservation and image yielding (4%–10% in 0.1 M phosphate buffer (PB), pH 7.4). The segments corresponding to the mammalian lumbosacral enlargement (dorsal segments 8–10 and sacral segments 1 and 2, Mortin and Stein, 1990) were dissected out (total length = 0.7–1 cm) and sectioned (70 μm thick) with a vibrating microtome in the transverse plane. The sections were rinsed in PB several times and then incubated during 48 h in the primary antibodies diluted in PB and 0.3% Triton X-100 for immunohistochemistry or PB and 0.004% saponin for immuno-transmission electron microscopy (TEM). For BrdU detection the sections were pre-treated with 2 N HCl for 30 min and then rinsed 10 times. After rinsing, the sections were incubated in secondary antibodies conjugated with fluorophores (Life Technologies, 90 min) or horseradish peroxidase (HRP, Jackson; 48 h). In double-labeling experiments we used Alexa 488 and Alexa 633 (Life Technologies) to avoid bleed-through. HOECHST (Life Technologies) was used for nuclear staining. Fluorescence signals were obtained using a confocal microscope (Olympus VF 300) and acquired with FluoView 5 software (Olympus).

For TEM, HRP-conjugated secondary antibodies were revealed with 3′,3′-diaminobenzidine. After checking the quality of the staining with a light microscope, the sections were post-fixed in 1% OsO_4_ (diluted in PB) and further processed as already described (Rehermann et al., 2009, 2011). Series of ultrathin sections mounted on Formvar-coated one-hole grids (2 × 1 mm) were analyzed with an electron microscope (JEOL X100). The immunostained cells appeared marked with an electron dense precipitate.

### Statistical Analysis

To assess the distribution of the 5-HT+ cells along the lumbar segments in both ShI (*n* = 6) and ScI (*n* = 6), the whole region was serially sectioned and stained cells counted on 10 sections of each animal (representing approximately 10% of the total). For statistical validation the non-parametric Mann-Whitney “U” test was applied at *p* < 0.05.

## Results

### Serotonergic Innervation in the Injured Spinal Cord

**Figure 2:**
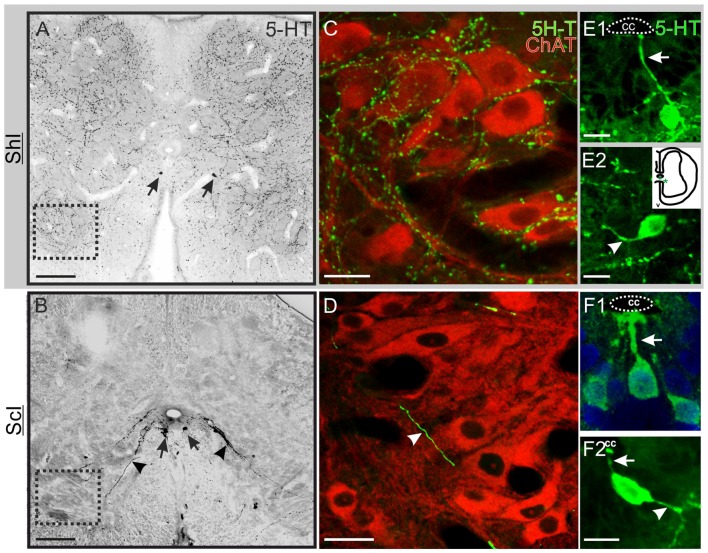
Serotonin (5-HT) innervation in the lumbar spinal cord. **(A)** Transverse section of the spinal cord of a sham-injured (ShI) turtle showing an extensive dense network of 5-HT+ varicose fibers and synaptic bouton-like structures. Few 5-HT+ cell bodies were found (arrows). **(B)** Section from an injured turtle (ScI) showing the pattern of 5-HT expression 30 days after injury, with conspicuous 5-HT+ cells (arrows) and fibers running towards the ventral horns (arrowheads). **(C,D)** show co-labeling of anti-ChAT antibody (red) and 5-HT antibody (green) in the regions outlined with a dotted lined square in **(A,B)**, respectively. **(C)** In the ShI group both the soma and proximal dendrites of ChAT+ motoneurons (Mns) are impinged by 5-HT+ bouton-like dots. **(D)** In the ScI group there are few thick processes crossing the Mns region (arrow head). **(E)** Some 5-HT+ cells in the gray commissure of the ShI spinal cord presented an apical process that reached the CC lumen (**E1**, arrow) whereas other cells bearing short processes were located further away from the CC (inset; **E2**, arrow head). **(F)** 5-HT+ cells from injured spinal cords showed both apical (**F1,F2**, arrows) and distal processes (**F2**, arrowhead). Scale bars: **(A,B)** = 100 μm; **(C,D)** = 20 μm; **(D,F)** = 10 μm.

To test putative changes in serotonergic innervation after SCI, we analyzed by means of immunohistochemistry the presence of serotonergic fibers and cells in the lumbar enlargement of ShI and ScI turtles (Figure 2). We found that the extrinsic descending serotonergic innervation in ShI animals (Figure 2A) was similar to that described previously in normal turtles (Kiehn et al., 1992). However, the extensive network of 5-HT+ fibers decreased dramatically 10 days after the SCI (not shown), virtually disappearing 30 days after spinal cord transection (Figure 2B). To explore the changes induced by SCI on the 5-HT innervation of Mns we performed double immunolabeling for choline acetyltransferase (ChAT) and 5-HT. In the ShI group the cell body and proximal dendrites of Mns were covered by a dense network of 5-HT+ varicosities (Figure 2C). However, in injured turtles, neither the dense fiber network nor the synaptic buttons were found (Figure 2D). Few isolated 5-HT+ processes were seen in the Mn pool area of injured animals (Figure 2D, arrowhead).

**Figure 3:**
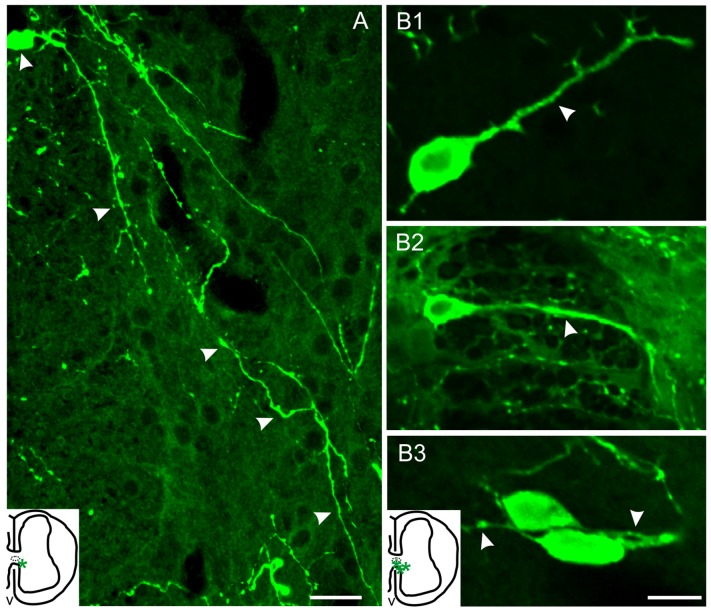
Serotonergic cells of injured turtles located near the CC (asterisks, inset drawings). **(A)** Cell with a long 5-HT+ process that can be followed along the ventral horn almost reaching the spinal surface (arrow heads). **(B1–3)** 5-HT+ cells with neuron-like morphology and with processes of variable-lengths (arrowheads). Scale bar: **(A)** = 20 μm, **(B)** = 10 μm.

**Figure 4:**
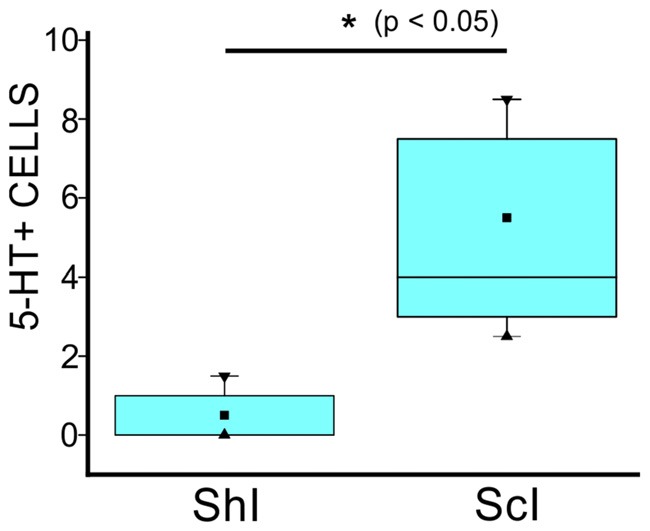
Quantification of 5-HT+ cells in the gray commissure and dorsal innermost portions of the ventral horns without contact with the central canal lumen of the lumbar enlargement of ShI and ScI turtles, 30 days after injury. (*p < 0.05). ■ MEAN; ▂ max/min; ▼ 99%; ▲ 1%.

Two distinct 5-HT+ cell populations were observed both in ShI and ScI animals: (1) a subpopulation of cells in the gray commissure with a single apical process contacting the lumen of the central canal (ShI, Figure 2E1; ScI, Figures 2F1,F2). These cells resembled cerebrospinal fluid-contacting neurons (CSFcNs) previously described in the spinal cord of turtles (Fernández et al., 2002; Russo et al., 2004; Reali et al., 2011). The number of this type of cells in ShI and ScI animals (30 days after SCI) was not statistically different (data not shown); and (2) a 5-HT+ cell population defined by cell bodies located in the gray commissure or dorsal innermost portions of the ventral horns and by the lack of contact with the lumen of the central canal (Figures 2B, 3A,B1–3). These serotonergic cells were scarce in control animals and had delicate thin processes (Figures 2A,E2). A quantitative analysis revealed that 30 days after SCI there was a significant increase (*p* < 0.05) in this type of 5-HT+ cells when compared to ShI (Figure 4). In contrast to ShI animals, the 5-HT+ cells that appeared in ScI animals showed relatively thick and long dendritic-like processes that run along the ventral horn, sometimes reaching the spinal surface (Figures 2B, 3A).

### Are New 5-HT+ Cells Born After SCI?

**Figure 5:**
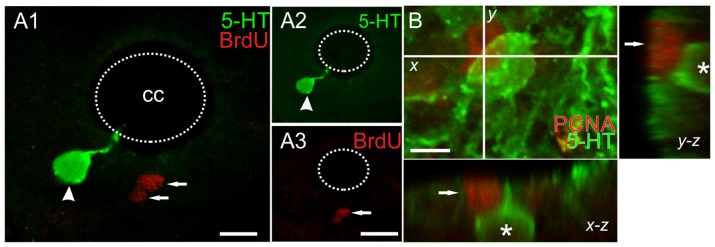
Double labeling with anti-5-HT and the proliferating cell markers **(A)** BrdU and **(B)** proliferating cell nuclear antigen (PCNA). **(A)** 5-HT+/BrdU- cell (arrowhead) and other BrdU+ nuclei (arrows). The green and red channels for the merged image in **(A1)** are shown in **(A2)** and **(A3)**, respectively. **(B)** Image of a 12 μm z-stack showing a 5-HT+ cell (asterisk) and one PCNA+ nucleus (arrow). Scale bar: **(A)** = 10 μm, **(B)** = 5 μm.

The CC in turtles is a neurogenic niche that generates new neurons in the postnatal life (Fernández et al., 2002). Therefore, our first goal was to determine whether the observed 5-HT+ cell increase was due to cell proliferation or to changes in the molecular phenotype of pre-existing non-5-HT cells. To address this problem we employed two proliferating-cell markers: BrdU and the proliferating cell nuclear antigen (PCNA). BrdU was injected as described (Figure 1C) in both ShI and ScI turtles. BrdU-labeled nuclei in the caudal segments of the spinal cord showed no significant differences between ShI and ScI animals (data not shown). In no case, were 5-HT+ cells with BrdU+ nuclei found (Figures 5A1–3). In addition, although we found cells with PCNA+ nuclei in different spinal cord regions there was no co-localization of PCNA and 5-HT labeling (Figure 5B). These findings indicate that the new 5-HT+ cells were not generated from the proliferation of spinal progenitors after SCI but instead via a molecular change in their original phenotype.

### Neuronal Nature of New Serotonergic Cells

**Figure 6:**
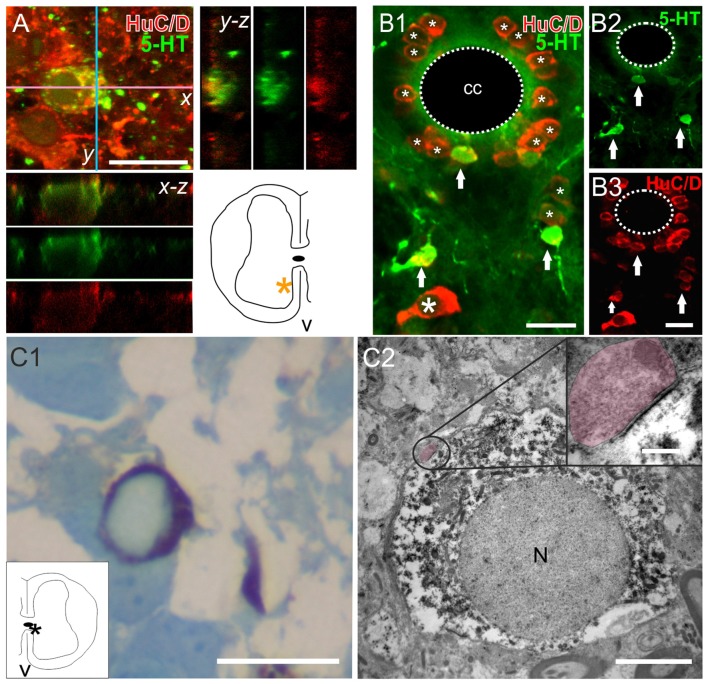
The neuronal nature of 5-HT+ cells in the spinal cord. **(A)** Double-labeling experiments to detect 5-HT and the neuronal marker HuC/D in a ShI turtle. Stack of a double-labeled cell and accompanying orthogonal planes. The inset in the lower right shows the location of the 5-HT+ cell (asterisk). **(B)** Images from an injured spinal cord showing the 5-HT+/HuC/D+ cells (**B1–3**, arrows). Notice that many HuC/D+ cells did not express 5-HT (**B1**, asterisks). **(C)** Semithin section of a 5-HT+ cell (revealed with DAB) stained with boracic methylene blue **(C1)**. The inset shows the location of the cell (asterisk). The same cell is shown in **(C2)** with transmission electron microscopy (TEM). Note the synaptic ending on the perikaryon encircled in the main panel and enlarged in the upper right inset (shaded in pink). Pre and post synaptic densities are clearly visible. Scale bar: **(A,B)**, 15 μm; **(C1)**, 15 μm, **(C2)**, main panel, 4 μm, zoom up, 75 nm. N, nucleus.

Another important matter is to know whether the new serotonergic cells belong to the neuronal lineage. To tackle this issue, we employed two technical approaches: (a) immunohistochemistry for the neuron-specific antigen HuC/D (Szabo et al., 1991); and (b) 5-HT immunostaining with TEM to reveal the presence of synaptic contacts onto re-specified serotonergic cells. We found that the two main kinds of 5-HT+ cells (CSFcNs and those in the ventral gray) expressed HuC/D. These features were found in the spinal cords of both ShI (Figures 6A) and injured animals (Figures 6B1–3). TEM analysis of 5-HT+ cells (Figure 6BC1) found in spinal cord-injured turtles revealed typical synaptic endings with well stained post-synaptic densities (Figure 6C2, main panel and inset) supporting their neuronal nature.

### Spinal Serotonergic Neurons Express Key Transcription Factors

**Figure 7:**
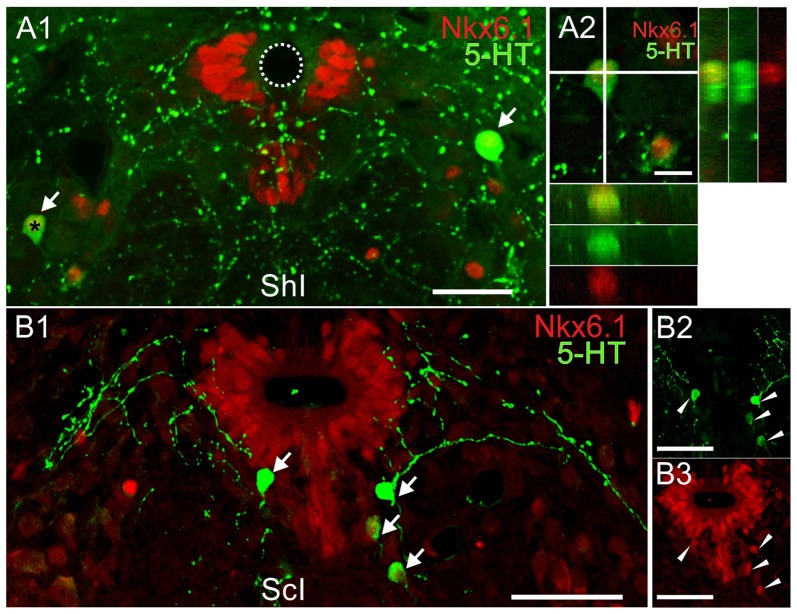
Co-labeling of the transcription factor (TF) Nkx6.1 and 5-HT in the lumbar enlargement of ShI and injured turtles. **(A)**5-HT (green) and Nkx6.1 (red) reactive cells in a sham-injured spinal cord (**A1**, arrows). The orthogonal planes **(A2)** of the cell marked by an asterisk in **(A1)** shows its Nkx6.1+ nucleus.**(B)** Double-labeled cells from an injured spinal cord (**B1**, arrows).The 5-HT (green) and Nkx6.1 (red) immunoreactivity for the cells in **(B1)** is shown in **(B2)** and **(B3)**, respectively (arrowheads). Scale bar: **(A1,B1–3)** = 50 μm; **(A2)** = 10 μm.

The expression of specific TF such as Nkx6.1 and Nkx2.2 is known to be involved in the differentiation of serotonergic neurons (Briscoe et al., 1999). We found Nkx6.1+ nuclei localized mainly in the lateral and ventral domains of the CC (Figures 7A1,B1). They were also sparingly present in the ventromedial gray and ventral horns (Figures 7A1,B1, arrows) all along the rostro-caudal axis (data not shown). In the lateral domains of the CC the distribution of the Nkx6.1+ nuclei was similar to BLBP+/Pax6+ progenitors previously described (Russo et al., 2008).

5-HT+ neurons expressed Nkx6.1 (Figures 7A1,2,B1–3) in the ShI (20 of 21 cells) and ScI animals (120 of 125 cells) suggesting that in both conditions the synthesis of 5-HT involves an Nkx6.1-dependent pathway.

**Figure 8:**
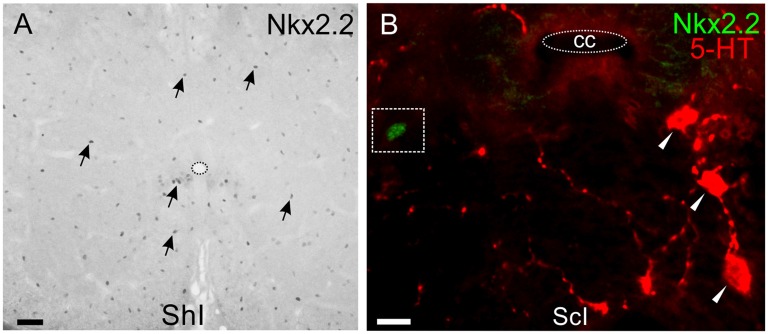
Nkx2.2 expression in lumbar spinal cord. **(A)** Nkx2.2+ nuclei show homogeneous distribution in the gray and white matter of a ShI cord (arrows). **(B)** Nkx2.2 expression in a lumbar section of the ScI turtle is similar to normal pattern and does not co-localize in 5-HT+ cells (red). Scale bar: **(A)** = 40 μm; **(B)** = 10 μm.

As Nkx2.2 is a TF involved in the generation of oligodendrocytes and serotonergic neurons, we then explored its expression both in the normal and injured spinal cord. Nkx2.2+ nuclei are ubiquitous both in the gray and white matter of the uninjured spinal cord (Figure 8A) and probably belong to oligodendrocyte progenitors (Fu et al., 2002). However, none of those nuclei corresponded to 5-HT+ neurons, neither in ShI (76 cells) nor in ScI turtles (72 cells; Figure 8B).

### Are 5-HT_1A_ Receptors Involved in Respecification of 5-HT+ Cells?

**Figure 9:**
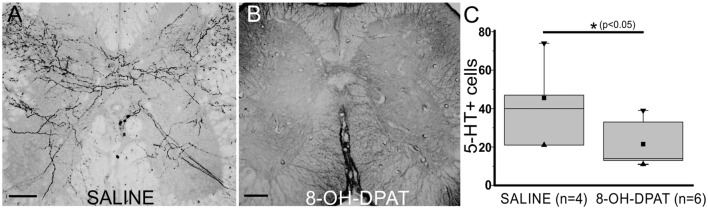
5-HT_1A_r are involved in neuronal respecification. 5-HT immunoreactivity in lumbar sections of non-treated (**A**, saline) and (±)-8-Hydroxy-2-dipropylaminotetralin hydrobromide (**B**, 8-OH-DPAT)-treated turtles 10 days after injury. **(C)** Plot showing a statistical difference in the number of 5-HT+ cells (*p* < 0.05) in control (saline) and 8-OH-DPAT treated animals 10 days after SCI. ■ MEAN, ▂ max/min, ▼ 99%, ▲ 1%. Scale bar: **(A,B)** = 50 μm.

Previous work showed that 5-HT_1A_r activation inhibited neuronal differentiation in the hippocampus (Diaz et al., 2013). In order to study a possible role of 5-HT_1A_r in inhibiting neuron respecification, we treated spinal cord-injured turtles with the selective 5-HT_1A_ agonist 8-OH-DPAT or saline for 10 days (Figures 9A,B). We found that there were significantly fewer 5-HT+ neurons in 8-OH-DPAT-treated turtles than in non-treated animals (Figure 9C).

## Discussion

The interruption of descending serotonergic innervation by SCI leads to various events that disrupt motor activity (Ghosh and Pearse, 2015). We show here that in the turtle, SCI induces an increase in the number of spinal serotonergic neurons below the lesion that likely compensates for the loss of descending serotonergic input.

### 5-HT Innervation Shifts From Extrinsic- to Spinally-Generated in Response to Injury

Both extrinsic and intrinsic serotonergic neuromodulation is exerted on neural circuits (Marder, 2012). Whereas serotonergic innervation arising from spinal neurons predominates in primitive vertebrates such as lampreys (Van Dongen et al., 1985; Barreiro-Iglesias et al., 2008) most serotonergic modulation in fish (Ritchie and Leonard, 1982; McLean and Fetcho, 2004), turtles (Kiehn et al., 1992) and mammals (Jordan et al., 2008) relies on fibers descending from the brainstem. Local spinal serotonergic innervation shares common features among vertebrates as 5-HT-containing neurons in fish, turtles (this study), rats (Newton et al., 1986) and monkeys (Lamotte et al., 1982) are relatively scarce and located ventral to the central canal in lamina X. The dramatic reduction of 5-HT terminals observed after SCI in most vertebrates (Ghosh and Pearse, 2015) was associated in turtles with an increase in the number of serotonergic neurons below the lesion. A spinal serotonergic system seems to account for the 5-HT+ terminals that remain after spinal cord transection in the elasmobranch fish (Ritchie et al., 1984), although it is not clear whether there is upregulation of spinal-generated innervation. In goldfish (Takeda et al., 2008) and zebrafish (Kuscha et al., 2012), SCI induces a several-fold increase in the number of spinal 5-HT neurons which is restricted to the lesion site. During regeneration of the spinal cord in zebrafish, the increased number of 5-HT neurons is later pruned to reach numbers similar to control animals (Kuscha et al., 2012). There is less information regarding a possible plasticity of a spinal 5-HT system in mammals. Interestingly, it has been recently shown that cells containing the enzyme L- amino acid decarboxylase acquire the ability to produce 5-HT provided its precursor 5-HTP is supplied to rats with a lesion at the level of the second sacral segment (Wienecke et al., 2014). This raises the possibility that mammals have some elements of the plasticity of the spinal serotonergic system displayed by some non-mammalian vertebrates.

### Mechanisms of 5-HT Spinal System Plasticity

The increased number of 5-HT neurons in goldfish (Takeda et al., 2008) and zebrafish (Kuscha et al., 2012) results from the generation of new neurons via active cell proliferation of progenitor-like cells in the lesion site. SCI in turtles induces an increase in cell proliferation which is spatially restricted to the lesion epicenter (Rehermann et al., 2011). In line with this, we found that a lesion at a low thoracic level did not affect the rate of cell proliferation in the lumbar enlargement. In addition, the fact that we did not find 5-HT cells labeled with BrdU or PCNA indicates that, unlike in fish, the new 5-HT cells in turtles do not arise from neurogenesis. Instead, our results suggest that new 5-HT cells result from neurotransmitter respecification of pre-existing cells as has been shown in the developing spinal cord of Xenopus (Dulcis and Spitzer, 2008; Demarque and Spitzer, 2010). Although the immunohistochemistry for HuC/D showed the neuronal nature of new 5-HT cells, it is not clear from our study whether the cells respecified to 5-HT neurons belonged already to the neuronal lineage or were undifferentiated cells.

The specification of raphe 5-HT neurons during development requires signals such as sonic hedgehog and genetic programs involving TF such as Foxa2, Ascl1, Nkx2.2 and Nkx6.1 (Smidt and van Hooft, 2013). The transcription factor Nkx2.2 seems to be sufficient to start 5-HT specification and together with Ascl1 drive the expression of Lmx1b and Pet1 needed for terminal differentiation and maintenance of the 5-HT phenotype (Pattyn et al., 2004; Gaspar and Lillesaar, 2012). However, the transcriptional programs seem to be diverse because there are Pet1- independent 5-HT neurons in the forebrain of zebrafish (Gaspar and Lillesaar, 2012) and in Nkx2.2 mutants a subset of 5-HT neurons in the hindbrain is spared (Jensen et al., 2008). In contrast to the abundant information on the molecular mechanisms that generate 5-HT neurons in the brainstem and forebrain, little is known about the transcriptional programs that determine spinal serotonergic cells. After SCI in turtles, an increase in 5-HT neurons occurred in the dorso-medial aspect of the ventral horn over a territory where a subset of cells expressed Nkx6.1. Because the production of serotonergic neurons in rhombomere 1 needs a high level of expression of Nkx6.1 (Briscoe et al., 1999; Craven et al., 2004; Kiyasova and Gaspar, 2011) it is tempting to speculate that Nkx6.1 cells in the ventral horn of the turtle spinal cord represent a cellular subpopulation permissive for neurotransmitter respecification after injury. In addition to Nkx6.1, the transcription factor Nkx2.2 is also needed during the early stages of serotonergic differentiation (Gaspar and Lillesaar, 2012; Smidt and van Hooft, 2013). Despite widespread expression of Nkx2.2, 5-HT neurons in the spinal cord of the turtle did not express this transcription factor either before or after SCI. In other vertebrates, Nkx2.2 is only briefly expressed during development (Briscoe et al., 1999; Cheng et al., 2003) so it may be possible that we missed the time window in which Nkx2.2 is expressed in neurons respecified to a 5-HT phenotype. Another possibility is that the transcriptional program in the postnatal spinal cord is different from that of the developing hindbrain (Jensen et al., 2008) and Nkx2.2 is not required for differentiation towards a 5-HT phenotype. Future studies should address the details of the molecular programs implied in serotonergic respecification in the turtle spinal cord.

Activity-dependent neurotransmitter respecification has been thoroughly characterized in Xenopus (Spitzer, 2012). Lowering the electrical activity in the hindbrain of Xenopus embryos, produced an increase in tryptophan hydroxylase and 5-HT-containing neurons by the activation of Lmx1b as a result of the decreased frequency of Ca^2+^ spikes (Demarque and Spitzer, 2010). Similarly, SCI in the turtle would lead to a reduction in the activity of spinal circuits below the lesion and may turn on the genetic programs starting the production of 5-HT in permissive Nkx6.1 cells. Whether electrical activity and calcium signaling play a role in 5-HT respecification of spinal cord cells in the turtle remains to be experimentally tested.

A possible trigger for respecification could be the loss of 5-HT background activation of 5-HT receptors in Nkx6.1 cells due to degeneration of descending serotonergic fibers. In fact, blockade of 5-HT_1A_r in embryonic medulla-spinal cord organotypic cultures induces an increase in 5-HT+ cells (Branchereau et al., 2002). Our results suggest that this mechanism is also at work in the spinal cord of turtles because activation of 5-HT_1A_r with specific agonist 8-OH-DPAT suppressed the increase in 5-HT cell number. It would be interesting to explore whether as descending 5-HT fibers regenerate (Rehermann et al., 2009) the increased activation of 5-HT_1A_r reverses the respecified neurons to their original phenotype. The time window analyzed in this study precluded the exploration of this possibility.

### Functional Significance of 5-HT Respecification After SCI

Neurotransmitter respecification after SCI may have important implications for functional recovery. It has been proposed that both in the developing and adult nervous system there are reserve pool neurons that can switch their neurotransmitter to do a new “job” thereby adjusting the output of neural networks to different functional demands or pathological conditions (Dulcis and Spitzer, 2012). Within this context, the respecification of pre-existing cells in the turtle spinal cord below the lesion may take over the job of supra-spinally mediated serotonergic modulation, contributing to the functional recovery described in these amniote vertebrates (Rehermann et al., 2009). Our electron microscopic data showed that 5-HT neurons receive synaptic contacts on their processes suggesting they are functionally integrated within local spinal circuits. Although new 5-HT neurons send processes that invade the ventral horn reaching the Mn pool, no 5-HT terminals were observed around Mns. This raises the possibility that 5-HT signaling from these new serotonergic neurons may be via volume conduction (Ridet et al., 1993). The transplantation of embryonic serotonergic cells below the level of spinalization in rats improves postural and locomotor activity (Gimenez Y Ribotta et al., 1998), indicating that even artificial restoration of spinal serotonergic modulation (likely via paracrine action) can help functional recovery. The synthesis of 5-HT by aromatic amino acid decarboxylase from 5-HTP is responsible for the increased excitability of Mns in the sacral cord of spinalized rats (Wienecke et al., 2014). Overall, these studies suggest that local spinal serotonergic modulation in vertebrates can effectively change the excitability of spinal circuits partly compensating for the loss of extrinsic modulation. The understanding of the mechanisms and functional impact of serotonergic respecification in response to SCI in an amniote vertebrate with regeneration capabilities and substantial functional recovery (Rehermann et al., 2009) may provide clues to design novel therapeutical strategies.

## Author Contributions

The study was done in the department of Neurofisiología Celular y Molecular, Instituto de Investigaciones Biológicas Clemente Estable. GF: conception and design of the experiments; collection, analysis and interpretation of immunohistochemical and anatomical data; writing of the manuscript. MIR: animal surgery, collection of immunohistochemical and anatomical data. CA: collection of immunohistochemical and anatomical data. OT-C: electron microscopy studies, drafting of the manuscript. RER: conception and design of the experiments, analysis and interpretation of data, writing of the manuscript. All authors approved the final version of the manuscript.

## Conflict of Interest Statement

The authors declare that the research was conducted in the absence of any commercial or financial relationships that could be construed as a potential conflict of interest.
